# The 10‐year treatment outcome of open dialogue‐based psychiatric services for adolescents: A nationwide longitudinal register‐based study

**DOI:** 10.1111/eip.13286

**Published:** 2022-03-25

**Authors:** Tomi Bergström, Jaakko Seikkula, Birgitta Alakare, Mia Kurtti, Päivi Köngäs‐Saviaro, Elina Löhönen, Jouko Miettunen, Hannele Mäkiollitervo, Jyri J. Taskila, Katriina Virta, Kari Valtanen

**Affiliations:** ^1^ Department of Psychology University of Jyväskylä Jyväskylä Finland; ^2^ Faculty of Health and Sport University of Agder Kristiansand Norway; ^3^ Department of Psychiatry Länsi‐Pohja Healthcare District Kemi Finland; ^4^ Center for Life Course Health Research University of Oulu Oulu Finland; ^5^ Medical Research Center Oulu Oulu University Hospital and University of Oulu Oulu Finland

**Keywords:** child and adolescent psychiatry, cohort study, cost‐effectiveness, family therapy, long‐term follow‐up

## Abstract

**Aim:**

To evaluate the 10‐year treatment outcomes and cost‐effectiveness of adolescents' mental health treatment initiated under the social network‐oriented open dialogue (OD) approach.

**Methods:**

This longitudinal register‐based study included all persons who, for the first time, received psychiatric treatment in Finland during the period 1 January 2003–31 December 2008, and who were aged 13–20 at onset (*n* = 44 868). The OD group included all persons whose treatment commenced in the Western Lapland catchment area (*n* = 780), this being the only region in Finland where OD covered the entire mental healthcare service at the time of inclusion. The comparison group (CG) included the rest of Finland (*n* = 44 088). The primary outcome variables were *psychiatric treatment* and/or *disability allowances* at the end of the 10‐year follow‐up, or *death*. The secondary outcome variables were *treatment and disability expenses*. Generalized linear models weighted by inverse probability of treatment were used to study the association between OD and the primary outcomes. Population proportions were used to evaluate the cost‐effectiveness of the approaches.

**Results:**

Treatment that commenced outside OD was associated with higher odds of continuing to receive treatment (adjusted odds ratio [aOR] 1.4; 95%CI 1.2–1.6) and disability allowances (aOR 1.6; 95%CI 1.2–2.1) at the end of the 10‐year follow‐up. No significant difference in the mortality ratio emerged. The cumulative 10‐year expenses per capita were lower under OD.

**Conclusions:**

OD associated with favourable long‐term outcomes, but due the observational design and possible residual confounding, further studies with a more robust research design are required.

## 1 INTRODUCTION

Because mental health problems typically emerge early in life, there has been growing interest in the early management and multidisciplinary integration of services for young people with mental health difficulties (McGorry & Mei, 2018). Open dialogue (OD) is one example of an approach involving the integration of existing services, seeking to guarantee immediate need‐adapted response and continuity of care for service users and their families (Seikkula et al., 2006). OD has demonstrated promising outcomes in the treatment of first‐episode psychosis, especially in the Finnish Western Lapland region, where the approach originated, and where the entire public psychiatric service was systematically reorganized to support a network‐oriented and immediate dialogical response to all mental health crises in the region (Bergström et al., 2018; Seikkula et al., 2006; Seikkula et al., 2011). The promising results have subsequently been reported beyond the Western Lapland region (Bouchery et al., 2018; Buus et al., 2019; Gordon et al., 2016; Granö et al., 2016; Tribe et al., 2019), but information is still limited concerning the entire service‐level outcome and cost‐effectiveness of the OD approach (Freeman et al., 2019).

The aim of this longitudinal register‐based study was to evaluate the 10‐year treatment outcomes and cost‐effectiveness of the treatment that commenced in services based on the need‐adapted and social network‐oriented OD approach.

## 2 METHOD

### 2.1 Study design

Data were obtained from Finnish national social and healthcare registers covering all adolescents aged 13–20 who (for the first time) received psychiatric treatment in Finland during the period 1 January 2003–31 December 2008 (*n* = 44 868). Information was gathered from registers up to the end of the year 2018, thus enabling a continuous 10‐year follow‐up for all persons in the study. A detailed description of the data sources is provided in the Supporting Information (Table S1).

To address the main goal, we examined long‐term treatment outcomes from psychiatric treatment that commenced in the Western Lapland catchment area, this being the only region in Finland where OD covered the entire regional mental healthcare service for adolescents at the time of inclusion (Seikkula et al., 2011). The main findings were compared with the rest of Finland (standard care).

Previous research indicates that OD could enable a more individualized integration of existing methods and services in efforts to address a difficult life situation in a need‐adapted manner, simultaneously helping people with mental health difficulties to maintain their sense of agency and social functioning (Bergström, 2020). Based on this, we hypothesized that treatment that commenced in OD‐based mental health services for adolescents would be associated with a lesser use of (i) mental health services and (ii) mental health disability allowances at the end of the 10‐year follow‐up, as compared to treatment that commenced in standard adolescent psychiatric care. We also hypothesized that (iii) OD would be associated with a decreased mortality ratio, as compared to standard care.

### 2.2 Catchment area and treatment approach

Finland is a Nordic country with a homogeneous population of 5.5 million. The Finnish mental healthcare system is publicly funded, and the municipalities are responsible for providing services to all their residents. Adolescents who need psychiatric care are usually referred from primary and/or school healthcare services to a secondary healthcare system, operating under the 21 Finnish hospital districts funded by a consortium of municipalities.

The Western Lapland catchment area consists of the south‐western parts of Finnish Lapland. The population of the area had decreased from 68 557 in 2000 to 61 172 in 2018, reflecting the national trend towards urbanization. Since the 1980s, the local mental healthcare system in Western Lapland area has been gradually reorganized in such a way as to make a dialogical and network‐oriented response possible for all mental crises in the region.

Following the regional municipalization of healthcare services in the early 2000s, the local adolescent psychiatric clinic continued to operate under the hospital district, and to deliver OD‐based services for all residents in the Western Lapland region aged 13–20 years. Treatment in the adolescent clinic was initiated by contact with the local 24/7 psychiatric emergency services, and/or directly with any of the mental healthcare staff. No referrals were needed.

The primary aim in the OD treatment was to gather all relevant people together in joint network treatment meetings as soon as possible. The meetings often took place in at people's homes or in another place of their own choice. Family members and/or other relevant people of the patient's choice (e.g. from school) were invited to participate in joint network treatment meetings, in which all decisions and interpretations were made within reciprocal dialogues. At least two clinicians participated in the network treatment meeting, and all clinicians, irrespective of their professional background and work unit, were equally responsible for ensuring both the continuity of care and a dialogical response to the crisis. Psychiatric diagnoses were not regularly used, and the primary aim was the integration of care, based on the current needs of individuals and their social networks. Psychiatric medication was used mainly as a last resort, to support other kinds of care.

According to Olson, Seikkula, & Ziedonis (2014), the OD approach has two fundamental features: (1) It is a community‐based, integrated treatment system that make an effort to engage families and social networks from the very start of their search for help. (2) Dialogical practice within the network treatment meetings constitutes the key therapeutic context of OD. In this way, OD‐based services differ from more conventional mental health service provision, in which care is usually organized and delivered at the level of the individual, and is based predominantly on the diagnosis and immediate symptom reduction in line with predetermined group‐level treatment guidelines (Razzaque & Stockmann, 2016; World Health Organization, 2021).

At the time of the inclusion years, OD‐based services were provided for all adolescents and young adults who used mental health services in Western Lapland (Seikkula et al., 2011). Furthermore, 90% of all staff members in the adolescent clinic had received 3‐year on‐the‐job psychotherapist training, tailored specifically to support the main principles of OD. The training was funded via regular in‐service training funds granted for Finnish hospital districts. As there were no adolescent psychiatric wards in the catchment area, hospital care for minors was arranged in collaboration with the University Hospital of Oulu.

### 2.3 Sampling

#### 2.3.1 Open dialogue group

The OD group was formed from all persons whose treatment contact with the mental health services occurred at the ages of 13–20 in the Western Lapland region, within the inclusion period 1 January 2003–31 December 2008 (*n* = 1171). We excluded from the sample all cases who had received mental health treatment prior to onset in an adolescents' clinic (*n* = 391; 33%). This was because the primary goal was to study first‐contact patients, and because there was a lack of information on how systematically OD‐based services had been delivered in the local child psychiatric clinic at the time of the inclusion.

#### 2.3.2 Comparison group

The comparison group (CG) was formed on the basis of similar inclusion criteria to those of the OD group. Thus, we first detected all adolescents aged 13–20 who had received mental healthcare treatment outside the Western Lapland region in the years 1 January 2003–31 December 2008 (*n* = 67 712). From this sample, we excluded all cases who had received mental health treatment prior to the onset of adolescent psychiatric treatment (*n* = 23 624; 34%).

Since diagnoses were not regularly applied under OD, we were unable to match cases based on diagnostic distributions. Nevertheless, at population level within Finland, regional differences in the prevalence of psychiatric diagnoses among adolescents were small at the time of inclusion (Table S2). Hence, unregistered diagnostic distribution (i.e. symptom expressions) should follow similar distributions when all cases are included.

Note, that Northern Finland has traditionally presented a higher prevalence rate for severe mental health problems as compared to the rest of the Finland (e.g. Perälä et al., 2008), and these problems are unlikely to remain unreachable in a small region with a low‐threshold mental health service. Hence, there should not be any under‐representation of severe mental health crises in the OD group, even if diagnoses were not formally used or registered. This is supported by earlier study, in which the incidence of first‐episode psychosis in Western Lapland was aligned with the rest of Finland when standard diagnostic procedures were used for research purposes (Bergström, 2020).

Despite this, given the low‐threshold service, it is possible that in the OD group there was over‐representation of the *milder* mental health problems that are not regularly referred to the mental health services. In our primary analyses we used the annual incidence of *first‐contact patients* and the *length of treatment* to further evaluate and adjust for this.

It should also be noted that there are regional variations in how adolescents' psychiatric services are organized in Finland. This means that in this study, the comparison group represented standard mental healthcare only at a general level.

### 2.4 Variables

The onset time was defined as the first entry in the adolescents' clinic. The data extended as far as death, or else a point 3651 days from onset. The baseline and primary outcome variables were formed by combining information from different registers (Table 1).

**TABLE 1:** Baseline and primary outcome variables

Baseline variables	Description
Age	Age at the time of the first register entry in psychiatric care
Gender	Male/female
Foster care placements	Yes: if there were one or more foster care placements prior to onset
Days in foster care	Summary of days for all foster care placements prior to onset
General hospital visits	Number of visits to non‐psychiatric units, prior to onset
General hospital days	Number of days spent in non‐psychiatric hospitals, prior to onset
Short treatment	Yes: if there were three or fewer visits to psychiatric units in the first treatment year
Primary outcome variables	
Mental health treatment at the end of the follow‐up	Yes: if there were one or more visits to specialized or primary mental healthcare units, and/or psychiatric hospital days, and/or psychiatric medication purchases, between days 3284 and 3651 from onset (i.e. in the last follow‐up year)
Mental health disability allowances at the end of the follow‐up	Yes: if in the last follow‐up year there were one or more payments of full or partial disability allowances, or cash rehabilitation benefits, granted due to psychiatric disorders
Death	Yes: if the patient had died during follow‐up

Secondary outcome variables were formed to evaluate the cost‐effectiveness of OD. Hospital days and outpatient visits were converted into euros on the basis of the municipal invoicing prices (annually predetermined) of the Western Lapland catchment area in 2021. As there was a lack of detailed information on outpatient treatment and treatment costs, we used a regular outpatient price (of 187 euros per visit) to create an estimation of the total costs of outpatient care. A sensitivity analysis was conducted by calculating all outpatient meetings under OD at a regular price for network treatment meetings and/or home visits with two or more clinicians (290 euros per visit).

Expenses on medication purchases, sickness allowances, and basic social assistance were obtainable directly from registers. All disability allowances were converted into euros by using the cost of Finnish guaranteed pension (838 euros per month).

### 2.5 Statistical methods

Outliers were detected and trimmed by using Tukey fences. *U*‐test and Chi‐squared test were used to compare group differences. Observable differences in baseline variables were adjusted via a stabilized inverse probability of treatment weighting (SIPTW) (Austin & Stuart, 2015).

Multivariable logistic regression was used to calculate the propensity scores on how baseline variables predicted the probability of treatment. The regression model included all the baseline variables. For the analysis of the treatment outcome at the end of the 10‐year follow‐up, propensity scores were further adjusted for the loss caused by deaths during the follow‐up.

Propensity scores were used to calculate the SIPTWs for each case. Weighted generalized linear models with binomial probability distribution and a logit link function were then used to test the main hypotheses. Sensitivity analyses with *E*‐value (Marthur et al., 2018) were conducted to examine the extent to which unmeasured confounders would cause a significant ratio above 1.0 to be non‐significant.

Cumulative 10‐year expenses were calculated for each case. For comparative purposes, a population proportion was used, dividing the expenses incurred by the population of adolescents of the same age at the time of the inclusion year, in Western Lapland (OD), and in the rest of Finland (CG). The absolute and population‐based proportions of 10‐year expenses for each inclusion year are presented in Supporting Information (Tables S3 and S4).

All *p* values <0.05 were considered statistically significant. Statistical analyses were conducted via IBM SPSS Statistics 26 for Windows.

## 3 RESULTS

### 3.1 Sample characteristics

The mean age at the time of onset was 16.1 years in OD and 16.3 years in CG (Table 2). In OD, 68% of the patients were female, while in CG the figure was 64%.

**TABLE 2:** Demographic and clinical characteristics prior to and after weighting

	Non‐weighted sample			Stabilized inverse probability of treatment‐weighted sample		
	OD *n =* 780	CG *n =* 44 088	*p*	OD *n =* 762	CG *n =* 44 105	*p*
Baseline characteristics						
Age (mean [sd])	16 (2)	16 (2)	0.01	16 (2)	16 (2)	0.6
Gender, female	68%	64%	0.01	64%	63%	0.6
Foster care (yes)	6%	8%	0.02	8%	8%	0.6
Days in foster care (mean [sd])	59 (380)	70 (387)	0.4	75 (426)	70 (387)	0.7
General hospital visits (mean [sd])	4 (5)	5 (6)	0.2	5 (6)	5 (6)	0.4
General hospital days (mean [sd])	2 (2)	1 (2)	0.01	1.4 (2)	1.4 (2)	1
Short treatment	38%	29%	0.01	29%	29%	0.9
Primary outcomes at the end of the 10‐year follow‐up						
Death	1.3%	1.6%	0.4	1.2%	1.6%	0.3
Suicide	<0.6%	0.8%	0.2	<0.6%	0.8%	0.2
Treatment contact	30%	39%	<0.001	31%	39%	<0.001
Disability allowance	7%	11%	<0.001	7%	11%	<0.001
Secondary outcomes in the 10‐ year follow‐up						
Hospital days (mean [sd])	7 (32)	20 (59)	<0.001	7 (34)	20 (59)	<0.001
Outpatient visits (mean [sd])	26 (33)	34 (39)	<0.001	28 (34)	34 (40)	<0.001
Disability expenses (mental health) (mean [sd])	3627 (11522)	5522 (14842)	<0.001	4061 (12280)	5517 (14835)	<0.001
Disability expenses (other) (mean [sd])	786 (1790)	699 (1708)	0.16	748 (1762)	700 (1708)	0.4
Basic social assistance expenses (mean [sd])	7854 (12389)	8116 (13407)	0.56	8454 (12876)	8108 (13400)	0.5
Medication expenses (mental health) (mean [sd])	286 (652)	640 (1011)	<0.001	293 (651)	640 (1011)	<0.001
Medication expenses (other) (mean [sd])	1823 (1946)	2396 (2178)	<0.001	1896 (2012)	2396 (2178)	<0.001

Abbreviations: OD, Open Dialogue group; CG, Comparison group; SD, Standard deviation.

In OD, only 5% of the patients received a psychiatric diagnosis during the first year from onset, while in CG the figure was 65%. Over the entire 10‐year follow‐up, 33% of the OD group and 78% of the CG group had one or more register entries with a psychiatric diagnosis. As compared to the CG, the OD group showed significantly smaller diagnosis proportions (cases with a diagnosis per 1000 people of the same age within the catchment area) as follows: for any F diagnosis, 35 vs. 66; for F20–F29, 4 vs. 8; for F30–F39, 20 vs. 38; and for F40–F48,19 vs. 33. As compared to the CG, those in the OD group who had received a psychiatric diagnosis during follow‐up were more likely to have received medication (83% vs. 74%, *p* < .001), and disability allowances (54% vs. 38%, *p* < .001). The figures are in line with the main premise of OD‐based services, in which psychiatric diagnoses are used mainly in prolonged crises, where they may be required for the sake of obtaining medication reimbursements and disability allowances.

Despite the low prevalence of psychiatric diagnoses, the overall annual incidence of new patients was higher in OD than in CG (18/1000 vs. 15/1000 persons of the same age). However, when only those cases with more than three outpatient visits during their first treatment year were included, the annual incidence of new cases evened out (10/1000 vs. 10/1000). We used this cut‐off to adjust for very short and/or consultative treatment contacts, since these were potentially over‐represented in OD due to the lack of referral services.

### 3.2 Primary outcome

At the end of the follow‐up, more people from CG than from OD were still receiving psychiatric treatment and disability allowances due to mental health disorders. No difference was found in mortality and suicide rates (Table 2). The administration of treatment outside OD predicted ongoing treatment and disability allowances at the end of the follow‐up (Table 3). *E*‐values indicated that it would require moderate‐to‐substantial confounding to render the findings non‐significant.

**TABLE 3:** Primary long‐term outcomes under OD as compared to standard care

Treatment commenced outside OD catchment area	Odds ratio	95% CI	*p*	E‐value for effect estimate^a^	E‐value for lower CI limit^b^
Death	1.4	0.7–2.8	0.3		
Treatment contact	1.4	1.2–1.6	<0.001	1.7	1.4
Disability allowances	1.6	1.2–2.1	<0.001	2.6	1.7

^a^ The minimum strength of association on the risk ratio scale that an unmeasured confounder would need to possess in order to fully explain away the observed association.

^b^ *E*‐values for the 95% CI limit closest to the null denote the minimum strength of association on the risk ratio scale that an unmeasured confounder would need to possess in order to shift the 95% CI to include the null value.

### 3.3 Cost analysis

In CG, the average costs of psychiatric treatment and mental health disability allowances were significantly higher than in OD. In OD, the total and average basic social assistance expenses and other disability expenses were higher, but not at a statistically significant level (Table 2). The cumulative 10‐year cost of all new adolescent patients who came to the treatment during one calendar year was 439 euros per capita in OD and 539 euros per capita in CG (Table 4). When all the outpatient visits under OD were calculated at a higher price the figure was 485 euros per capita (see sensitivity analysis in Section 2.4).

**TABLE 4:** Average 10‐year expenses (euros) for all adolescent patients who came to the treatment during one calendar year (divided by the population of people of the same age in each inclusion year)

	Open dialogue group		Comparison group	
	Mean^a^ (euros)	95%CI	Mean^a^ (euros)	95%CI
Hospital treatment	87	41–133	201	181–221
Outpatient treatment	93	75–111	91	78–104
Psychiatric medication^b^	3	2–4	8	7–8
Other medication	33	26–39	34	32–36
Disability allowances (mental health)	66	37–95	79	61–97
Disability allowances (other)	14	11–17	10	8–13
Basic social security	142	109–176	116	100–133
Total	439 euros per capita	354–524	539 euros per capita	503–575

*Note*: Information is lacking on the rehabilitative psychotherapy provided by private psychotherapists; this was substantially more common in the years 2013–2018 in other parts of Finland than in the Western Lapland catchment area (18/1000 vs. 4/1000 people aged 19–30) (SII, 2021).

^a^ Mean of six inclusion years (2003–2008).

^b^ Anatomical Therapeutic Chemical (ATC) classification codes: N05–N06.

## 4 DISCUSSION

Treatment that commenced under OD‐based services was associated with a lesser use of psychiatric services and mental health disability allowances in the 10‐year follow‐up. No differences in the mortality ratio were observed. Total basic social security expenses were higher in OD; however, since there were no statistically significant differences in the average social security payments, this could be due to the relatively higher number of patients in the OD group, and to particular regional factors. For example, job and education opportunities in Finland have been centralized to the larger regions, and the unemployment rates are higher in Western Lapland than in many other parts of Finland (THL, 2021).

Nevertheless, it was noticeable that even though there were relatively more people receiving treatment under OD, the total 10‐year costs for the use of services and for disability allowances were still lower. In reality this difference is even more substantial, since we lacked information on psychotherapy delivered in the private sector, which was more common in other parts of Finland than in Western Lapland (SII, 2021).

Overall, the primary findings were in line with emerging evidence on the effectiveness of OD (Bergström et al., 2018; Bouchery et al., 2018; Buus et al., 2019), and with the idea that success in the early management of mental problems has the potential to transform outcomes and make care more cost‐effective (Uhlhaas et al., 2020). However, in the absence of more detailed information on the fidelity to OD practice, and also on the clinical characteristics of the patients, it remains a challenge to determine what elements of OD are associated with a specific outcome, and whether unidentified confounding factors might have influenced the results. The findings of this study nevertheless demonstrate how adolescent mental health treatment initiated in OD‐based services was associated with long‐term outcomes at group level, within the real‐world mental health service as a whole.

At the same time, it should be noted that the OD cohort, too, contained a proportion of people who made substantial use of available services and of disability allowances. Moreover, the lower usage of mental health services, and the savings in public expenses, do not necessarily translate to indirect treatment‐related benefits, including subjective experiences of wellbeing.

### 4.1 Strengths and limitations

Finnish registers are considered to constitute a reliable (Kiviniemi, 2014; Sund, 2012) and valid (Lahti & Penttilä, 2001; Sund, 2012) source of information. The registers also enabled the inclusion of all persons in Finland who received adolescent psychiatric treatment, indicating that the results offered ecologically valid information on real‐world psychiatric treatment. In this case, the usage of mental health services and disability allowances provided strong indications on the actual clinical and functional outcomes—bearing in mind that in Finland, health and social services are guaranteed to the entire population on the basis of statutory national social security provisions. It should nevertheless be noted that the registers were not originally designed for research purposes, and that inaccuracies could arise.

Unobservable regional differences could also have impacted on the findings. However, the population in Finland is homogeneous, and since the regional variations in e.g. socio‐economic and ethnic status are small, it is unlikely that these factors would cause significant residual confounding in this kind of register‐linked study (Kiviniemi, 2014). It is notable also that in a historical comparison study the region of Western Lapland was found to have poor treatment outcomes from severe mental disorders prior the implementation of OD (Aaltonen et al., 2011). Thus, it seems unlikely that there would be regional factors in Western Lapland that would explain the better treatment outcome under OD.

Nevertheless, given possible unobservable regional differences and other limitations in the data, one can speculate that the weighted population did not account for the totality of group differences, and that there could have been unobserved confounding factors. For example, we lacked a measurement of onset symptom severity, since diagnoses were not regularly used in OD. Moreover, within Western Lapland, the proportion of adolescents with any psychiatric diagnosis over the entire follow‐up was notably low, as compared to the high incidence of first‐contact patients.

Even though it is possible that the difference in observable diagnostic distributions reflect real regional differences in symptom severity (and hence influence the main findings), the results, and also earlier epidemiological data from the Western Lapland area (Bergström, 2020) indicate that the low prevalence of diagnoses is more likely to be due to the OD itself (within which psychiatric diagnoses tend to be used and registered only in cases of prolonged symptoms). Conversely, it is possible that success in the early management of mental health crises may itself reduce prolonged symptoms and thus the overall prevalence of psychiatric diagnoses.

The fact that most of the patients in the CG were diagnosed during their first treatment year is an indication of more conventional diagnostic practice, in which psychiatric diagnoses are used to guide the provision of mental healthcare services and treatment. This means that the registered diagnostic distributions were not comparable between samples, and that some unadjustable selection bias may have remained, even though we included nationwide all first‐contact adolescent patients in a specific time frame. Nevertheless, the annual incidence of new cases was consistent between CG and OD, and we were able to adjust for observable baseline characteristics.

In seeking to further compensate for potential sampling bias and overrepresentation of milder symptom cases in OD‐group, in conducting the cost analysis we used the absolute expenses divided by the population of the catchment area in each inclusion year. Since the proportions (relative to population) of the expenses were then partially independent of the proportions of patients with different symptom severities in OD and CG, cost‐analysis should provide valid and comparable information on total costs, even if there is an overrepresentation of milder cases in the OD group as compared to the comparison group.

Despite the adjustments applied, given the observational nature of this study and possible residual confounding, further studies with a more robust research design are required to produce information on the causality of the association between OD and the favourable mental health treatment outcomes observed.

## Supporting information


**Supplementary Table S1**Diagnostic prevalences among adolescents aged 13–17 in 21 Finnish hospital districts in the years 2008–2009; per 10 000 habitants; grouped by geographical location^1^

**Supplementary Table S2**Absolute cumulative 10‐year expenses (euros) for all new adolescent psychiatric patients per each inclusion year

**Supplementary Table S3**Per capita cumulative 10‐year expenses (euros) for all new adolescent psychiatric patient per each inclusion year

## Data Availability

The Health and Social Data Permit Authority Findata supervise the secondary use of all Finnish health and social care data including all of the data used in this study. Data can be accessed based on justified purposes. For more information and data permit applications see findata.fi.
