# Supplementary material for: The 10‐year treatment outcome of open dialogue‐based psychiatric services for adolescents: A nationwide longitudinal register‐based study
Source: Early Interv Psychiatry. 2022 Mar 25;16(12):1368–75. doi: 10.1111/eip.13286 (PMC10078679; doi:10.1111/eip.13286)
Supplement: Supplementary file 1 — Supplementary Table S1Diagnostic prevalences among adolescents aged 13–17 in 21 Finnish hospital districts in the years 2008–2009; per 10 000 habitants; grouped by geographical location1 [file EIP-16-1368-s001.docx]

**Online supplementary content**

| **Supplementary table 1. Data sources** | |
| --- | --- |
| Data source | Description |
| The Finnish Care Register of Health Care and the Register of Primary Healthcare, provided by the National Institute for Health and Welfare (THL) | Hospital admissions, outpatient treatment conducted in specialized healthcare units since 1998, and treatment conducted in the primary healthcare system since 2011. |
| Register of Child Welfare, provided by THL | Placements outside the home. |
| Basic social assistance register, provided by THL | Basic social assistance payments granted if the income does not cover essential daily expenses. |
| The register of disability pensions and reimbursed medicines, provided by the Social Insurance Institution of Finland | Full and partial disability allowance payments, cash rehabilitation benefit payments, and purchases of reimbursed medicines |
| The national cause-of-death register, provided by Statistic Finland | Death certificates issued by physicians, including the time and cause of death. |

| **Supplementary table 2. Diagnostic prevalences among adolescents aged 13–17 in 21 Finnish hospital districts in the years 2008–2009; per 10 000 habitants; grouped by geographical location^1^** | | | | | | | | |
| --- | --- | --- | --- | --- | --- | --- | --- | --- |
| Geographical area | South |  | West |  | East |  | North |  |
| Prevalence | Mean | sd | Mean | sd | Mean | sd | Mean | sd |
| Psychosis (F20–F29) | 1 | 0.5 | 0.6 | 0.5 | 1 | 1 | 1.2 | 1 |
| Mood disorder (F30–F39) | 9 | 3 | 8 | 3 | 10 | 1 | 8 | 3 |
| Neurotic disorders (F40–F48) | 8 | 1 | 7 | 3 | 7 | 2 | 7 | 3 |
| Behavioural disorders (F90–F98) | 9 | 3 | 8 | 3 | 7 | 1 | 7 | 4 |
| sd = standard deviation  ^1^Data derived from Finnish public data cubes provided by The National Institute for Health and Welfare (THL) | | | | | | | | |

| **Supplementary table 3. Absolute cumulative 10-year expenses (euros) for all new adolescent psychiatric patients per each inclusion year** | | | | | | |
| --- | --- | --- | --- | --- | --- | --- |
| Index year | 2003 | 2004 | 2005 | 2006 | 2007 | 2008 |
| **Open Dialogue group *(N*)** | **132** | **137** | **121** | **118** | **145** | **147** |
| Psychiatric hospital care | 288 826 | 469 775 | 792 852 | 673 341 | 833 753 | 395 035 |
| Mental health disability allowances | 418 669 | 402 527 | 492 820 | 218 206 | 517 804 | 780 064 |
| Basic social security expenses | 708 353 | 1 232 849 | 812 652 | 1 192 866 | 1 026 356 | 1 153 578 |
| Psychiatric medication expenses | 29 058 | 23 597 | 25 259 | 18 796 | 36 923 | 30 920 |
| Mental health outpatient care | 635 426 | 603 449 | 531 641 | 483 208 | 748 935 | 747 065 |
| Other disability allowances | 109 443 | 106 033 | 120 439 | 71 071 | 124 780 | 81 236 |
| Other medication expenses | 203 185 | 220 821 | 232 536 | 202 083 | 299 207 | 264 341 |
| **Comparison group *(N)*** | **7204** | **6986** | **6837** | **7319** | **7371** | **8371** |
| Psychiatric hospital care | 117 277 420 | 111 483 434 | 97 360 433 | 104 942 788 | 96 640 110 | 94 944 461 |
| Mental health disability allowances | 29 967 569 | 33 515 164 | 34 886 118 | 43 337 737 | 48 729 223 | 53 037 029 |
| Basic social security expenses | 52 841 013 | 52 352 515 | 53 233 373 | 61 305 222 | 63 168 690 | 74 916 989 |
| Psychiatric medication expenses | 4 065 381 | 3 625 910 | 3 523 632 | 3 888 178 | 3 733 674 | 4 107 123 |
| Mental health outpatient care | 41 528 586 | 40 430 335 | 42 142 881 | 47 754 190 | 49 943 773 | 58 501 267 |
| Other disability allowances | 6 968 806 | 6 121 835 | 5 130 330 | 4 802 358 | 3 945 245 | 3 872 127 |
| Other medication expenses | 17 614 616 | 16 830 443 | 16 615 559 | 18 033 777 | 17 313 600 | 19 258 372 |

| **Supplementary table 4. Per capita cumulative 10-year expenses (euros) for all new adolescent psychiatric patient per each inclusion year** | | | | | | |
| --- | --- | --- | --- | --- | --- | --- |
| Index year | 2003 | 2004 | 2005 | 2006 | 2007 | 2008 |
| **Open Dialogue group *(N*)** | **132** | **137** | **121** | **118** | **145** | **147** |
| Total N of persons aged 13–20 in Western Lapland catchment area | 7471 | 7318 | 7232 | 7136 | 7011 | 6825 |
| Psychiatric hospital care | 38 | 64 | 109 | 94 | 161 | 57 |
| Mental health disability allowances | 56 | 55 | 68 | 30 | 74 | 114 |
| Basic social security expenses | 95 | 168 | 112 | 167 | 146 | 169 |
| Psychiatric medication expenses | 3 | 3 | 3 | 2 | 5 | 4 |
| Mental health outpatient care | 85 | 82 | 108 | 68 | 106 | 109 |
| Other disability allowances | 15 | 14 | 16 | 10 | 17 | 12 |
| Other medication expenses | 27 | 30 | 32 | 28 | 42 | 38 |
| Total (euros per capita) | 319 | 416 | 448 | 399 | 551 | 503 |
| **Comparison group *(N)*** | **7204** | **6986** | **6837** | **7319** | **7371** | **8371** |
| Total N of persons aged 13–20 in the rest of Finland | 512 065 | 510 673 | 511 999 | 513 951 | 518 399 | 521 748 |
| Psychiatric hospital care | 229 | 218 | 190 | 204 | 186 | 182 |
| Mental health disability allowances | 59 | 66 | 68 | 84 | 94 | 102 |
| Basic social security expenses | 103 | 103 | 104 | 120 | 122 | 144 |
| Psychiatric medication expenses | 8 | 7 | 7 | 8 | 7 | 8 |
| Mental health outpatient care | 81 | 80 | 82 | 93 | 97 | 112 |
| Other disability allowances | 14 | 12 | 11 | 10 | 8 | 8 |
| Other medication expenses | 34 | 33 | 32 | 35 | 33 | 37 |
| Total (euros per capita) | 528 | 519 | 494 | 554 | 547 | 593 |
